# Numerical and Thermal Investigation of Magneto-Hydrodynamic Hybrid Nanoparticles (SWCNT-Ag) under Rosseland Radiation: A Prescribed Wall Temperature Case

**DOI:** 10.3390/nano12060891

**Published:** 2022-03-08

**Authors:** Ali Hassan, Azad Hussain, Mubashar Arshad, Meznah M. Alanazi, Heba Y. Zahran

**Affiliations:** 1 Department of Mathematics, University of Gujrat, Gujrat 50700, Pakistan; imbashrii@gmail.com; 2 Department of Physics, College of Science, Princess Nourah bint Abdulrahman University, P.O. Box 84428, Riyadh 11671, Saudi Arabia; mmalenazy@pnu.edu.sa; 3 Laboratory of Nano-Smart Materials for Science and Technology (LNSMST), Department of Physics, Faculty of Science, King Khalid University, P.O. Box 9004, Abha 61413, Saudi Arabia; heldemardash@kku.edu.sa; 4 Nanoscience Laboratory for Environmental and Biomedical Applications (NLEBA), Metallurgical Lab. 1, Department of Physics, Faculty of Education, Ain Shams University, Roxy, Cairo 11757, Egypt

**Keywords:** hybrid nanofluid, magneto-hydrodynamic rotating flow, kerosene oil, radiation

## Abstract

Thermal heat generation and enhancement have been examined extensively over the past two decades, and nanofluid technology has been explored to address this issue. In the present study, we discuss the thermal heat coefficient under the influence of a rotating magneto-hydrodynamic hybrid nanofluid over an axially spinning cone for a prescribed wall temperature (PWT) case. The governing equations of the formulated problem are derived by utilizing the Rivlin–Ericksen tensor and boundary layer approximation (BLA). We introduce our suppositions to transform the highly non-linear partial differential equations into ordinary differential equations. The numerical outcomes of the problem are drafted in MATLAB with the of help the boundary value problem algorithm. The influences of several study parameters are obtained to demonstrate and analyze the magneto-hydrodynamic flow characteristics. The heat and mass transfer coefficients increase and high Nusselt and Sherwood numbers are obtained with reduced skin coefficients for the analyzed composite nanoparticles. The analyzed hybrid nanofluid (SWCNT-Ag–kerosene oil) produces reduced drag and lift coefficients and high thermal heat rates when compared with a recent study for SWCNT-MWCNT–kerosene oil hybrid nanofluid. Maximum Nusselt (Nu) and Sherwood (Sh) numbers are observed under a high rotational flow ratio and pressure gradient. Based on the results of this study, we recommend more frequent use of the examined hybrid nanofluid.

## 1. Introduction

Progress in the growth of technologies describing the behavior of fluid flows over a cone or through a cone has increased vastly. The practical applications of this particular phenomenon have importance and applications in industries such as engineering, dental health, paper production, and solar power collection. The generation and absorption of heat with unsteadiness inflow over a cone can be applied in many engineering fields, such as in the petroleum industry, pharmaceutical processing, environment control, solar pumps, and plantations. Major industrial applications utilize hybrid nanofluids, such as in parabolic trough collectors, solar energy, food processing, and drug delivery [1,2,3,4,5].

Anilkumar and Roy discussed the mixed convection of unsteady flow over a rotating cone [6]. Hussain et al. [7] explored heat transportation over a rotating cone in MHD flow over a radiation regime with hybrid nanofluids. Nadeem and Saleem [8] studied magneto-hydrodynamic flow over a rotating cone for time-dependent mixed convection. Hanifa et al. [9] elaborated radiative hybrid nanofluids over a cone to examine the prescribed wall temperature and heat flux. Rajeswari and Nath [10] focused their study on overstretching surfaces to assess the unsteady flow of a rotating fluid. Nadeem and Saleem [11] described unsteady mixed convection for a second-grade rotating fluid over a cone. The cone configuration has been examined by numerous researchers [12,13,14,15].

Heat transfer using ordinary fluids such as water, ethylene glycol, and oils has been explored extensively over the past two decades. Natural convection is a significant phenomenon with numerous applications in geophysics, solar energy, nuclear energy, and electronic cooling. The single-phase model has its limitations, while the two-phase model is more trustworthy for heat transfer enhancement. Moreover, the range of experimental studies should be broadened to different sets of nanofluids to obtain solid outcomes, which will help understand the convective behavior of nanofluids [16].

Das et al. [17] provided an exceptional review on heat transfer with nanofluids, along with the thermophysical properties of nanofluids. Godson et al. [18] described the enhancement of heat transfer for different sets (combinations) of nanofluids. Dangthongsuk and Wongwises [19] elaborated convective heat transfer phenomena in nanofluids. Farajohalli et al. [20] focused on heat transfer in shells, tubes, and heat exchangers using nanofluids. Heat transfer utilizing nanofluid technology has been discussed and explored by a plethora of scientists, including several notable articles [21,22,23,24,25].

The recently emerged “hybrid nanofluids” contain two nanometer (nm)-sized (metallic or non-metallic) particles in a host base liquid. This category of fluids has created new possibilities for convective heat transfer. Heat transfer depends upon the properties of the base liquid and nanoparticles, and thermal conductivity plays a pivotal role. Fluids such as water, oils, and ethylene glycol have low thermal conductivity as compared to hybrid nanofluids. The correlations for different thermophysical properties have been developed in the past few years and tested experimentally. The obtained results have been astonishing, encouraging researchers to explore these new kinds of fluids more extensively. The industrial importance of hybrid nanofluids is unimaginable, as hybrid nanofluids enhance heat transfer rates, reduce skin friction, and reduce production costs [26,27,28].

Yang et al. [29] presented an updated review on the applications of hybrid nanofluids, their properties, their fabrication, and their environmental effects. Tulu and Ibrahim [30] discussed the use of hybrid nanofluids over a spinning cone with variable viscosity and thermal conductivity. Chirstov et al. [31] provided a broad review on soft hydraulics for compliant conduits from Newtonian to complex fluid flows. Tilili et al. [32] explored the effects of radiation on hybrid nanofluids over a rotating cone in a mutable permeable medium. Ghadikolae et al. [33] analyzed the use of mixture-based hybrid nanofluids over a rotating cone with shape factors. Gul et al. [34] elaborated the flow within the canonical gap between the cone and surface of a rotating disk using a hybrid nanofluid. Adamaki et al. [35] introduced a new kind of nanoparticle known as a super-paramagnetic nanoparticle (SPAN) and elaborated the manufacturing process, Structure, properties, simulations, and applications. Sheriff et al. [36] utilized hybrid nanofluids to discuss irreversibility effects in peristaltic motion with heat absorption. Sheriff et al. [37] investigated varying fluid features for water-based nanoparticles for heat and peristaltic propagation. Numerous researchers have utilized nanofluids and hybrid nanofluids to enhance the heat transfer coefficient, including several quality studies [38,39,40,41,42,43,44,45,46,47,48,49,50,51,52,53,54,55].

Following the above deep literature review, no other studies have presented this problem in this manner. The novelty of this is article is the discussion of the flow of a hybrid nanofluid (SWCNT-Ag/KO) over a spinning cone with thermal radiation and magnetic effect. This paper enables the reader to follow our investigation of the prescribed wall temperature (PWT). Firstly, a small concentration (0.05%) of (SWCNT) nanoparticles is dispersed in the host liquid, kerosene oil (KO), to obtain the SWCNT-KO nanofluid, then the SWCNT-Ag/KO hybrid nanofluid is achieved by adding 0.05% solid vol. fraction of silver (Ag) nanoparticles. The governing equations of the formulated problem are derived utilizing the Rivlin–Ericksen tensor and boundary layer approximation (BLA). We then introduce our hypotheses and obtain ordinary differential equations from highly non-linear partial differential equations. The numerical outcomes of the problem are drafted in MATLAB using a help boundary value problem algorithm. The impacts of different flow parameters are elaborated, and the heat transfer and mass coefficients are obtained and analyzed.

## 2. Statement of Problem

**Figure 1:**
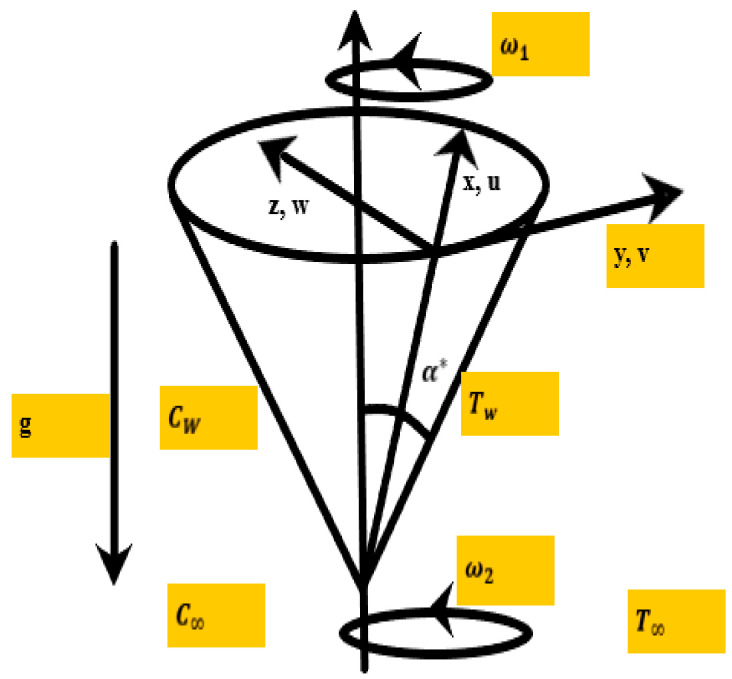
Physical physique and coordinates of the problem. *u*, *v*, *w* are components of velocity, *T_W_*, *C_W_* denotes temperature and concentration at wall, respectively. *T_∞_, C_∞_* are free stream temperature and concentration, respectively. *ω_1_* is cone’s rotation, *ω_2_* denotes fluid’s rotational velocity. *g* is gravitational force and α^*^ describes angle of rotation.

Let us consider a magneto-hydrodynamic viscous incompressible rotating hybrid nanofluid flow over a vertical spinning cone with radiation effects for the prescribed wall temperature case. The below-mentioned flow configuration $\omega_{1}\;\mathrm{and}\;\omega_{2}$ denote the cone and fluid’s rotational velocities, respectively, and $\alpha^{*}$ is the angle of rotation; u, v, w are components of velocity in the meridional segment (x-component), tangential path (y-component), and to the surface of the cone (z-component), respectively. The nanoparticles considered for this problem are single-walled carbon nanotubes (SWCNTs), and silver (Ag) kerosene oil is selected as the base liquid. The nanofluid is formed by distributing a small volume fraction 0.05% of silver nanoparticles into the base liquid. The desired hybrid nanofluid (SWCNT-Ag/KO) is achieved by distributing a different volume fraction of single-walled carbon nanotubes. Figure 1 below shows the physical physique and coordinates of the problem.

### 2.1. Flow Governing Equations

The model flow governing equations are an extension of the work by Hussain et al. [12,13,14]. Moreover, the complete methodology used to obtain the problem-solving equations is provided in [15]. The continuity, conservation of momentum, and energy are as [7] follows:

(1) $$\frac{x\partial u}{\partial x}+u+\frac{x\partial w}{\partial z}=0,$$

(2) $$\begin{array}{ll} \frac{u\partial u}{\partial x}+\frac{w\partial u}{\partial z}+\frac{\partial u}{\partial t} & -\frac{v^{2}}{x}\\ & =\nu_{hnf}\left(\frac{\partial^{2}u}{\partial z^{2}}\right)-\frac{v_{e}^{2}}{x}-\frac{\sigma B_{0}^{2}u}{\rho_{hnf}}+\zeta gcos\alpha^{*}\left(T-T_{\infty }\right)\\ & +g\zeta^{*}cos\alpha^{*}\left(C-C_{\infty }\right),\; \end{array}$$

(3) $$\frac{w\partial v}{\partial z}+\frac{u\partial v}{\partial x}+\frac{\partial v}{\partial t}+\frac{uv}{x}=\frac{\partial v_{e}}{\partial t}+\nu_{hnf}\frac{\partial^{2}v}{\partial z^{2}}-\frac{\sigma B_{0}^{2}v}{\rho_{hnf}},$$

(4) $$\frac{u\partial T}{\partial x}+\frac{w\partial T}{\partial z}+\frac{\partial T}{\partial t}=\alpha_{hnf}\frac{\partial^{2}T}{\partial z^{2}}-\frac{q_{r}}{{\left(\rho C_{p}\right)}_{hnf}}\frac{\partial^{2}T}{\partial z^{2}},$$

(5) $$\frac{\partial C}{\partial t}+\frac{u\partial C}{\partial x}+\frac{w\partial C}{\partial x}=D\frac{\partial^{2}C}{\partial z^{2}}.$$

The boundaries and initials conditions are as follows:

(6) $$\begin{array}{c} u\left(0,x,z\right)=u_{i},\;v\left(0,x,z\right)=v_{i},\;w\left(0,x,z\right)=w_{i}\;\;\;\;\;T\left(0,x,z\right)=T_{i},\;\\ \;C\left(0,x,z\right)=C_{i},\;\; \end{array}\},$$

(7) $$\begin{array}{c} u\left(t,x,0\right)=0,\;w\left(t,x,0\right)=0,\;v=\frac{\omega_{1}sin\alpha *}{\left(1-st\omega sin\alpha *\right)\;},\;\;T\left(t,x,0\right)=T_{w},\;\\ \;C\left(t,x,0\right)=C_{w}. \end{array}$$

The Rosseland approximation ($q_{r})$ expression is in the form of $q_{r}=-\frac{4\sigma^{*}}{3K^{*}}\frac{\partial T^{4}}{\partial y}$. This particular relation defines radiative heat flux. Here,$\;\sigma^{*}$ is the Stefan–Boltzmann constant and $K^{*}$ describes absorption. The assumed difference in temperature is quite small. Thus, the expansion of $T^{4}$ in terms of $T_{\infty }$ with the Taylor formulation is given below. The obtained expression will be truncated up to the first degree term and the truncation order is $O{\left(T-T_{\infty }\right)}^{2},$ which gives:

(8) $$T^{4}\approx 4TT_{\infty }^{3}-3T_{\infty }^{4},$$

Finally, the energy equation is obtained by utilizing the expression for the Rosseland approximation $q_{r}$ and energy equation (Equation (4)), then after substituting Equation (8) into the temperature equation (Equation (4)) we get our final energy equation:

(9) $$\frac{u\partial T}{\partial x}+\frac{w\partial T}{\partial z}+\frac{\partial T}{\partial t}=\alpha_{hnf}\frac{\partial^{2}T}{\partial z^{2}}+\frac{1}{{\left(\rho C_{p}\right)}_{hnf}}\frac{\partial^{2}T}{\partial z^{2}}\frac{16\sigma^{*}T_{\infty }^{3}}{3K^{*}},$$

We define the following transformation for the PWT case:

(10) $$\eta =\frac{z\;{\left(\omega sin\alpha^{*}\right)}^{0.5}}{\nu^{0.5}{\left(1-st\omega sin\alpha^{*}\right)}^{0.5}},\;v_{e}=\frac{x\omega_{2}sin\alpha^{*}}{\left(1-st\omega sin\alpha^{*}\right)},\;u\left(x,t,z\right)=-\frac{x\left(\omega sin\alpha^{*}\right)f^{\prime }\left(\eta \right)}{2\left(1-st\omega sin\alpha^{*}\right)},\;\;\alpha_{1}=\frac{\omega_{1}}{\omega },\;t^{*}=t\omega sin\alpha^{*},\;Pr=\frac{\nu }{\alpha },\;Sc=\frac{\nu }{D},\;\gamma_{1}=\frac{Gr_{1}}{{\left(Re_{L}\right)}^{2}},\;\gamma_{2}=\frac{Gr_{2}}{{\left(Re_{L}\right)}^{2}},\;N=\frac{\gamma_{2}}{\gamma_{1}},\;v=\frac{x\left(\omega sin\alpha^{*}\right)g\left(\eta \right)}{\left(1-st\omega sin\alpha^{*}\right)},\;w=\frac{{\left(\omega sin\alpha^{*}\right)}^{0.5}f\left(\eta \right)}{{\left(1-st\omega sin\alpha^{*}\right)}^{0.5}\nu^{0.5}},\;\;\;$$

$$\;\;G_{r_{1}}=\frac{cos\alpha^{*}\left(T_{0}-T_{\infty }\right)g\zeta L^{3}}{\nu^{2}},\;G_{r_{2}}=\frac{cos\alpha^{*}\left(C_{0}-C_{\infty }\right)g\zeta^{*}L^{3}}{\nu^{2}},\;\;\;C-C_{\infty }=\left(C_{w}-C_{\infty }\right)\varphi \left(\eta \right),\;T_{w}-T_{\infty }=\frac{x\left(T_{0}-T_{\infty }\right)cos\alpha^{*}}{L{\left(1-st\omega sin\alpha^{*}\right)}^{2}},\;C_{w}-C_{\infty }=\frac{x\left(C_{0}-C_{\infty }\right)cos\alpha^{*}}{L{\left(1-st\omega sin\alpha^{*}\right)}^{2}},\;T-T_{\infty }=\left(T_{w}-T_{\infty }\right)\theta \left(\eta \right),Re_{L}=\frac{\left(\omega sin\alpha^{*}\right)L^{2}}{\nu }.$$

The similarity transformations are utilized for Equations (2)–(5), while (1) is satisfied uniformly. Equations (2), (3), (5) and (9) will reduce to the following system of equations:

(11) $$\begin{array}{ll} f^{\prime \prime \prime }=2D_{1}H_{a}f^{\prime }- & D_{1}D_{2}\{2{\left(1-\alpha_{1}\right)}^{2}+2\gamma_{1}\left(\theta +N\varphi \right)+\left(\frac{1}{2}s\eta +f\right)f^{\prime \prime }+(s\\ & -\frac{1}{2}f^{\prime })f^{\prime }+2g^{2}\},\; \end{array}$$

(12) $$g^{\prime \prime }=D_{1}H_{a}g+D_{1}D_{2}\left\{\left(\frac{1}{2}s\eta +f\right)g^{\prime }-gf^{\prime }+s\left(g+\left(1-\alpha_{1}\right)\right)\right\},\;$$

(13) $$\theta^{\prime \prime }\left[\frac{D_{3}}{k_{f}}+\frac{4}{3}R_{a}\right]=PrD_{4}\left\{\frac{1}{2}s\eta \theta^{\prime }+2s\theta -\frac{1}{2}\theta f^{\prime }+\theta^{\prime }f\right\},$$

(14) $$\varphi^{\prime \prime }=Sc\left\{\frac{1}{2}s\eta \varphi^{\prime }+2s\varphi -\frac{1}{2}\varphi f^{\prime }+f\varphi^{\prime }\right\}.$$

Now, the boundaries for the PWT [2] case are given as:

(15) $$\{\begin{array}{c} f^{\prime }\left(0\right)=f\left(0\right)=0,\;g\left(0\right)=\alpha_{1},\;\theta \left(0\right)=\varphi \left(0\right)=1\\ f^{\prime }\left(\infty \right)=0,\;g\left(\infty \right)=1-\alpha_{1},\;\theta \left(\infty \right)=\varphi \left(\infty \right)=0.\; \end{array}$$

where η is the similarity variable; f, g, θ, and φ are dimensionless velocity components in the azimuthal and tangential direction, temperature, and concentration for the PWT, respectively. The Grashof numbers are represented by $G_{r_{1}},\;G_{r_{2}},\;$ while the buoyancy forces for temperature and concentration are represented by $\gamma_{1},\;\gamma_{2}$, respectively. The Grashof number ratio is N and *L* is the characteristic length. The ratio of the angular velocity of the cone to the composite angular velocity is presented by $\alpha_{1};$ Pr and Sc are the Prandtl and Sherwood or Schmidt numbers, respectively, while s describes the unsteadiness characteristic of the fluid.

### 2.2. Quantities of Physical Interest

The quantities of physical or engineering interest are the skin coefficients $\left(Cf_{x},\;Cf_{y}\right)$ in azimuthal and tangential directions, respectively, along with the Nusselt $\left(Nu_{x}\right)$ and Sherwood $\left(Sh_{x}\right)$ numbers, given as:

(16) $$Cf_{x}=\frac{\mu_{hnf}{\left[2\left(\frac{\partial u}{\partial z}\right)\right]}_{z=0}}{\rho_{f}{\left[\left(x\Omega sin\alpha^{*}\right){\left(1-st\Omega sin\alpha^{*}\right)}^{-1}\right]}^{2}},\;$$

(17) $$Cf_{y}=\frac{\mu_{hnf}{\left[2\left(\frac{\partial v}{\partial z}\right)\right]}_{z=0}}{\rho_{f}{\left[\left(x\Omega sin\alpha^{*}\right){\left(1-st\Omega sin\alpha^{*}\right)}^{-1}\right]}^{2}}.\;$$

(18) $$Nu_{x}=-\left\{\frac{k_{hnf}}{k_{bf}}+\frac{4}{3}R\right\}*\frac{{\left[\left(\frac{\partial T}{\partial z}\right)\right]}_{z=0}}{\left(T_{w}-T_{\infty }\right)},\;$$

(19) $$Sh_{x}=-\frac{{\left[\rho_{f}D\left(\frac{\partial T}{\partial z}\right)\right]}_{z=0}}{\left(C_{w}-C_{\infty }\right)},\;Re_{x}=\left(x\omega sin\alpha^{*}\right){\left(1-st\omega sin\alpha^{*}\right)}^{-1}/\nu .\;$$

In the dimensionless form, the physical quantities for the PWT are:

(20) $$Cf_{x}=-\left[\frac{R_{e_{x}}^{-\frac{1}{2}}f^{\prime \prime }}{D_{1}}\right]\;at\;\eta =0,\;Cf_{y}=-\left[\frac{R_{e_{x}}^{-\frac{1}{2}}g^{\prime }}{D_{1}}\right]\;at\;\eta =0,\;$$

(21) $$Nu_{x}=-\left[\left\{D_{3}+\frac{4}{3}R\right\}*Re_{x}^{-\frac{1}{2}}\theta^{\prime }\right],\;Sh_{x}=-\left[Re_{x}^{-\frac{1}{2}}\varphi^{\prime }\right]at\;\eta =0.$$

## 3. Numerical Solution

The governing flow equations of the problem are obtained with the help of the boundary layer approximation. The achieved highly non-linear PDEs are converted into a dimensionless set of equations for the PWT case. The set of new variables is introduced to generate the ODE. The problem is numerically tackled with the boundary value problem technique in MATLAB. The tolerance or convergence criterion of this particular solution is kept at 10^−6^. The set of our suppositions is as follow for the PWT case:

(22) $$f=y_{1},\;f^{\prime }=y_{2},\;f^{\prime \prime }=y_{3},\;f^{\prime \prime \prime }=y_{3}^{\prime },\;\;\;g=y_{4},\;g^{\prime }=y_{5},\;g^{\prime \prime }=y_{5}^{\prime },\;\;\theta =y_{6},\;\theta^{\prime }=y_{7},\;\theta^{\prime \prime }=y_{7}^{\prime },\;\;\;\;\varphi =y_{8},\;\varphi^{\prime }=y_{9},\;\varphi^{\prime \prime }=y_{9}^{\prime },\;\;\;a=\rho_{s1},\;b=\rho_{s2},\;c=\rho_{f},\;d={\left(\rho c_{p}\right)}_{f},\;\;e={\left(\rho c_{p}\right)}_{s1},\;j={\left(\rho c_{p}\right)}_{s2},\;k_{s1}=h,\;k_{s2}=q,\;k_{f}=r.\;\;\;$$

The ordinary differential equations for the PWT case are given as:

(23) $$\begin{array}{ll} y^{\prime }\left(3\right)=2(1-\varphi_{1}{)}^{2.5} & {\left(1-\varphi_{2}\right)}^{2.5}H_{a}y\left(2\right)-{\left(1-\varphi_{1}\right)}^{2.5}(1\\ & -{\varphi_{2})}^{2.5}\{\left(1-\varphi_{2}\right)\left[\left(1-\varphi_{1}\right)+\varphi_{1}\left(\frac{\rho_{s1}}{\rho_{f}}\right)\right]+\varphi_{2}\left(\frac{\rho_{s2}}{\rho_{f}}\right)\}[2{\left(1-\alpha_{1}\right)}^{2}\\ & +2\gamma_{1}\left(y\left(6\right)+Ny\left(8\right)\right)+\left(\frac{1}{2}s\eta +y\left(1\right)\right)y\left(3\right)+\left(s-\frac{1}{2}y\left(2\right)\right)\\ & +2(y\left(4{)}^{2}\right)],\; \end{array}$$

(24) $$\begin{array}{ll} y^{\prime }\left(5\right)=H_{a}{\left(1-\varphi_{1}\right)}^{2.5} & {\left(1-\varphi_{2}\right)}^{2.5}y\left(4\right)+{\left(1-\varphi_{1}\right)}^{2.5}(1\\ & {-\varphi_{2})}^{2.5}\left\{\left(1-\varphi_{2}\right)\left[\left(1-\varphi_{1}\right)+\varphi_{1}\left(\frac{\rho_{s1}}{\rho_{f}}\right)\right]+\varphi_{2}\left(\frac{\rho_{s2}}{\rho_{f}}\right)\right\}[\frac{1}{2}s\eta y\left(7\right)\\ & +y\left(1\right)y\left(5\right)-y\left(4\right)y\left(2\right)+sy\left(4\right)\left(1-\alpha_{1}\right)],\; \end{array}$$

(25) $$\begin{array}{ll} y^{\prime }\left(7\right)= & (\frac{K_{s2}+\left(n-1\right)K_{bf}-\left(n-1\right)\varphi_{2}\left(K_{bf}-K_{s2}\right)}{K_{s2}+\left(n-1\right)K_{bf}+\varphi_{2}\left(K_{bf}-K_{s2}\right)}\\ & \;\;\;*\frac{K_{s1}+\left(n-1\right)K_{f}-\left(n-1\right)\varphi_{1}\left(K_{f}-K_{s1}\right)}{K_{s1}+\left(n-1\right)K_{f}+\varphi_{1}\left(K_{f}-K_{s1}\right)}\\ & \;\;\;+\frac{4}{3}R{)}^{-1}\{(\left(1-\varphi_{2}\right)\left\{\left(1-\varphi_{1}\right)+\varphi_{1}\left[\frac{{\left(\rho C_{p}\right)}_{s1}}{{\left(\rho C_{p}\right)}_{f}}\right]\right\}\\ & \;\;\;+\varphi_{2}\left[\frac{{\left(\rho C_{p}\right)}_{s2}}{{\left(\rho C_{p}\right)}_{f}}\right])rPr(\frac{1}{2}\eta y\left(7\right)+2y\left(6\right)s-(\frac{1}{2}y\left(2\right)y\left(6\right)\\ & \;\;\;-y\left(7\right)y\left(1\right))\}\; \end{array}$$

(26) $$y^{\prime }\left(9\right)=Sc\left[(y\left(1\right)y\left(9\right)-\frac{1}{2}y\left(2\right)y\left(8\right)+2sy\left(8\right)+\frac{1}{2}s\eta y\left(9\right)\right].\;$$

The boundaries for the PWT are:

(27) $$\{\begin{array}{c} y\left(2\right)=y\left(1\right)=0,\;y\left(4\right)=\alpha_{1},\;y\left(6\right)=y\left(8\right)=1\\ y_{\infty }\left(2\right)=0,\;y_{\infty }\left(4\right)=1-\alpha_{1},\;y_{\infty }\left(6\right)=y_{\infty }\left(8\right)=0.\; \end{array}\;\;\;$$

The expressions $D_{1},\;D_{2},\;D_{3},\;\mathrm{and}\;D_{4\;}$ are defined as:

(28) $$D_{1}={\left(1-\varphi_{1}\right)}^{2.5}{\left(1-\varphi_{2}\right)}^{2.5},$$

(29) $$D_{2}=\left\{\left(1-\varphi_{2}\right)\left[\left(1-\varphi_{1}\right)+\varphi_{1}\left(\frac{\rho_{s1}}{\rho_{f}}\right)\right]+\varphi_{2}\left(\frac{\rho_{s2}}{\rho_{f}}\right)\right\},\;$$

$$\begin{array}{ll} D_{3}= & \frac{K_{s2}+\left(n-1\right)K_{bf}-\left(n-1\right)\varphi_{2}\left(K_{bf}-K_{s2}\right)}{K_{s2}+\left(n-1\right)K_{bf}+\varphi_{2}\left(K_{bf}-K_{s2}\right)}\\ & \;\;\;*\frac{K_{s1}+\left(n-1\right)K_{f}-\left(n-1\right)\varphi_{1}\left(K_{f}-K_{s1}\right)}{K_{s1}+\left(n-1\right)K_{f}+\varphi_{1}\left(K_{f}-K_{s1}\right)},\; \end{array}$$

(30) $$D_{4}=\left(\left(1-\varphi_{2}\right)\left\{\left(1-\varphi_{1}\right)+\varphi_{1}\left[\frac{{\left(\rho C_{p}\right)}_{s1}}{{\left(\rho C_{p}\right)}_{f}}\right]\right\}+\varphi_{2}\left[\frac{{\left(\rho C_{p}\right)}_{s2}}{{\left(\rho C_{p}\right)}_{f}}\right]\right).\;$$

## 4. Results and Discussion

We discuss the influence of distinct parameters, namely the rotational ratio, Hartman number, radiation parameter, and characterization of unsteadiness on the velocity, temperature, and concentration profiles. The skin frictions in azimuthal and tangential directions are obtained along with the Nusselt and Sherwood numbers. Table 1 presents the skin friction coefficients $\left(Cf_{x},\;Cf_{y}\right)$, heat transfer coefficient ($Nu_{x})$, and mass coefficient (Sc). Table 2 and Table 3 present the thermo-physical properties and relations utilized for this study, respectively. The comparison of present outcomes was established with previously published work in Table 4 and Table 5.

### 4.1. Velocity Profiles

**Figure 2:**
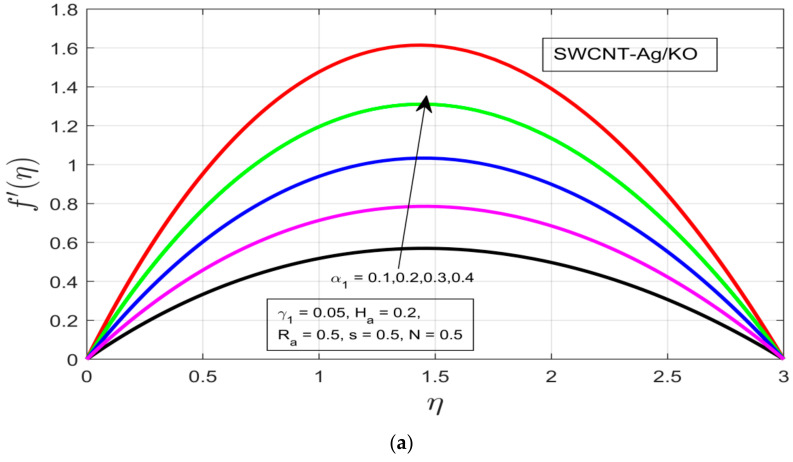

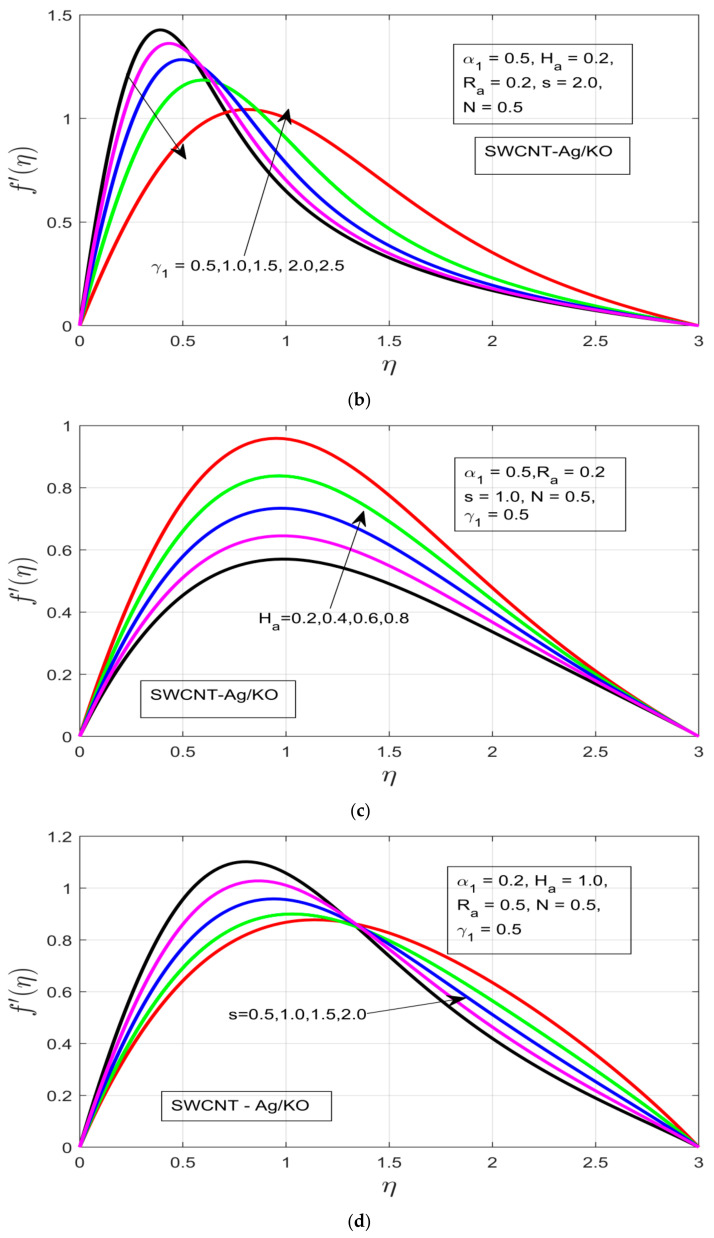
(**a**) Impact of the rotational ratio *α_1_* on *f′(η).* (**b**) Impact of the buoyancy parameter γ_1_ on *f′(η)*. (**c**) Impact of the Hartman number *H_a_* on *f′(η).* (**d**) Impact of the unsteadiness parameter *s* on *f′(η)*.

The influences of different study constraints $\left(\alpha_{1},\gamma_{1},\;H_{a},\;\mathrm{and}\;s\right)$ are discussed here on both profiles $f^{\prime }\left(\eta \right),g\left(\eta \right)$. Figure 2a–d demonstrates the influence of the rotational ratio, buoyancy parameter, Hartman number (magnetic field), and unsteadiness characterization parameter on $f^{\prime }\left(\eta \right)$. As the rotation $\alpha_{1}$ increases, an increment can be observed in the boundary layer thickness of the hybrid fluid motion (see Figure 2a) for the azimuthal velocity $f^{\prime }\left(\eta \right)$. The increased exerted pressure gradient $\gamma_{1}$, which acts as applied positive pressure, decreases the azimuthal velocity $f^{\prime }\left(\eta \right)$ resulting from the decline in the thermal boundary layer of the magnetized fluid (see Figure 2b). Figure 2c shows the impact of the Hartman number $H_{a}$ on the azimuthal velocity $f^{\prime }\left(\eta \right)$, where the increasing magnetic field generates enough Lorentz force to oppose the fluid motion but a contrast pattern is visible for the selected hybrid nanofluid (SWCNT-Ag/KO), while the temperature boundary enlarges under the increasing magnetic field $H_{a}$. Figure 2d shows that the impact of the unsteadiness parameter (s) on $f^{\prime }\left(\eta \right)$ the azimuthal profile decreases with increasing unsteadiness in the fluid behavior, resulting in a decrease in the thermal boundary layer of the flow motion.

**Figure 3:**
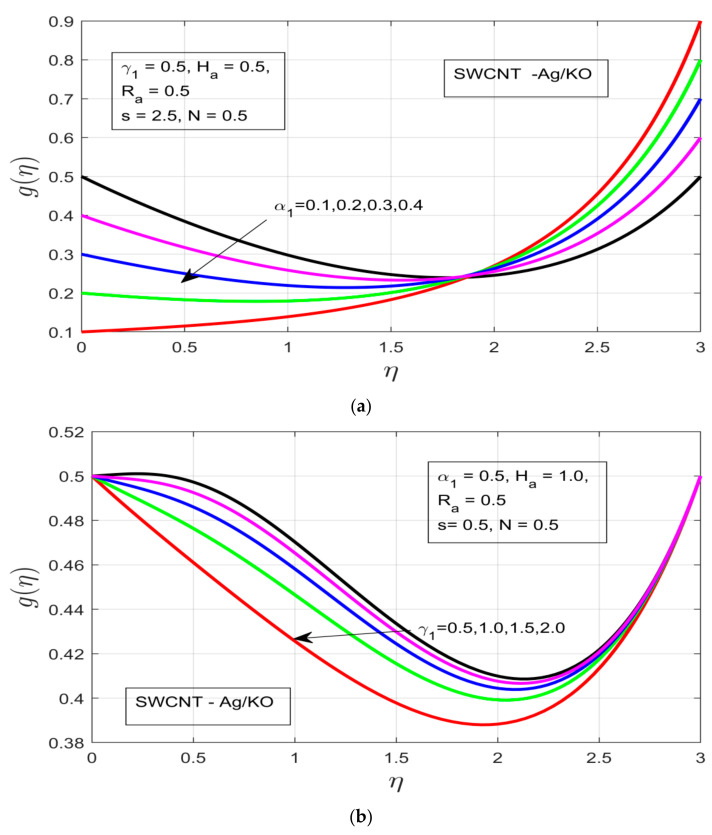

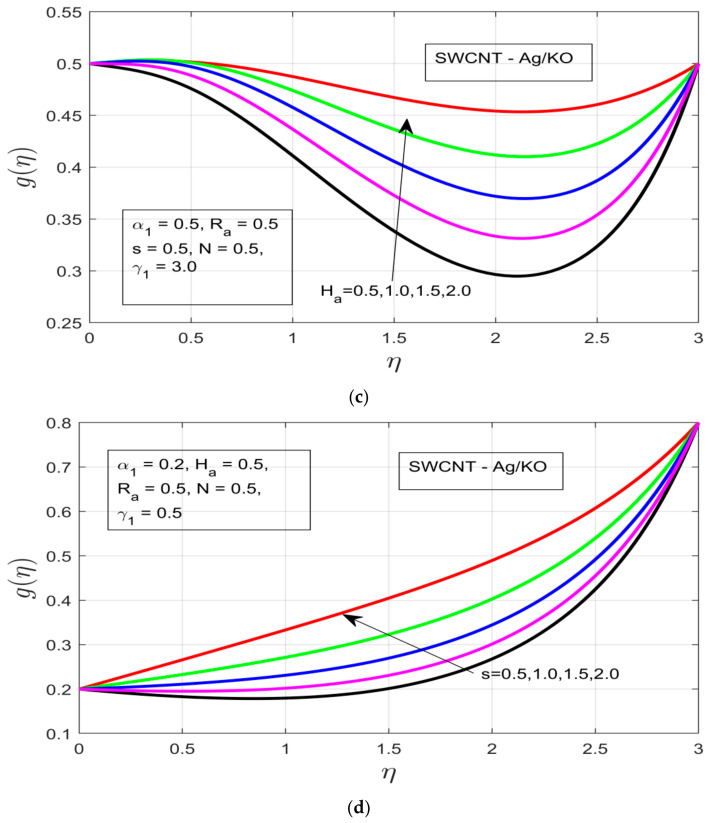
(**a**) Impact of the rotational ratio *α_1_* on *g(η)*. (**b**) Outcome of the buoyancy parameter *γ_1_* on *g(η)*. (**c**) Impact of the Hartman number *H_a_* on *g(η)*. (**d**) Impact of the unsteadiness parameter s on *g(η)*.

The influences of different study parameters $\left(\alpha_{1},\gamma_{1},\;H_{a},\;\mathrm{and}\;s\right)$ on the tangential profile of velocity g(η) are elaborated in Figure 3a–d. Figure 3a shows the influence of the rotational ratio $\alpha_{1}$ on the tangential velocity g(η), whereby the velocity profile is enlarged, resulting in an increased temperature boundary layer in the respective direction despite the presence of highly resistive Lorentz force due to the high magnetic field. Figure 3b covers the buoyancy parameter $\gamma_{1}$, whereby the pressure exerted with help of the buoyancy parameter decreases the thermal boundary layer, which happens due to the presence of high resistance in the fluid motion, which does not let the concentration layer move smoothly. Figure 3c shows the effects of the increasing magnetic field $H_{a}$ on the tangential velocity profile g(η), whereby the thermal boundary increases under increasing magnetized motion of the fluid. In Figure 3d, the tangential velocity g(η) increases with increasing fluid characterization (s), which allows an increment in the thickness of the thermal boundary layer, even under high buoyancy force and magnetic impact.

### 4.2. Temperature and Concentration Profiles

**Figure 4:**
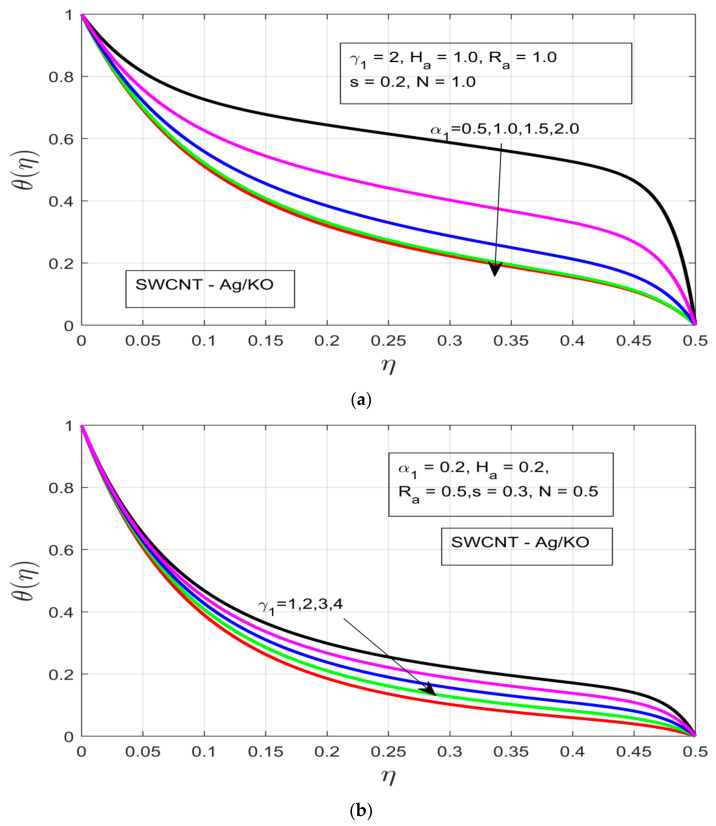

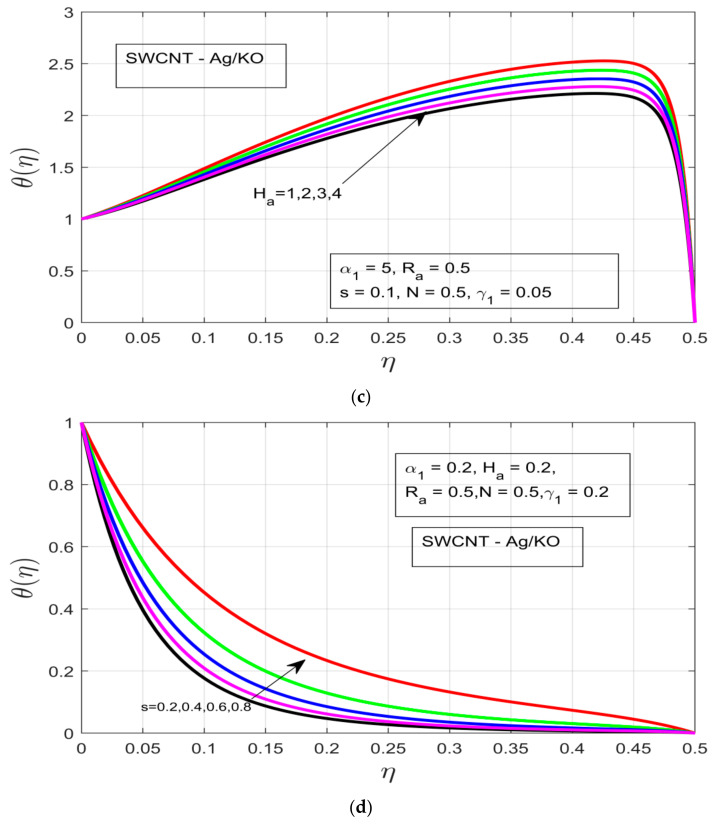
(**a**) Influence of the rotational ratio *α_1_* on the temperature profile *θ(η)*. (**b**) Impact of the buoyancy parameter *γ_1_* on the temperature profile *θ(η)*. (**c**) Impact of the Hartman number *H_a_* on the temperature profile *θ(η)*. (**d**) Impact of the unsteadiness parameter *s* on the temperature profile *θ(η)*.

The behavior of the thermal and concentration layers are observed under the influence of distinct study constraints $\left(\alpha_{1},\gamma_{1},\;H_{a},\;R_{a},\;\mathrm{and}\;s\right)$. The temperature profile θ(η)  can be observed in Figure 4a–d, whereby the increments in the rotational ratio $\alpha_{1}$ and buoyancy force $\gamma_{1},$ decrease the thermal boundary layer associated with flow motion. The increasing rotational ratio $\alpha_{1}$ decreases the thermal boundary layer more drastically as compared to the increment in buoyancy force $\gamma_{1}$, which is due to the high resistance caused by the high-pressure gradient, as can be seen in Figure 4a,b, respectively. The impact of the Hartman number $H_{a}$ is analyzed in Figure 4c, where under the increasing $H_{a}$ thermal boundary layer it increases over time, although as the flow motion encounters the presence of a strong Lorentz force, which acts as the resistive force, it immediately drops as a result of the drastically decreased thermal boundary. Figure 4d shows the influence of the unsteadiness parameter (s) on the temperature profile θ(η), whereby the temperature profile θ(η) decreases with an increase in unsteadiness in the free stream velocity of the flow motion.

**Figure 5:**
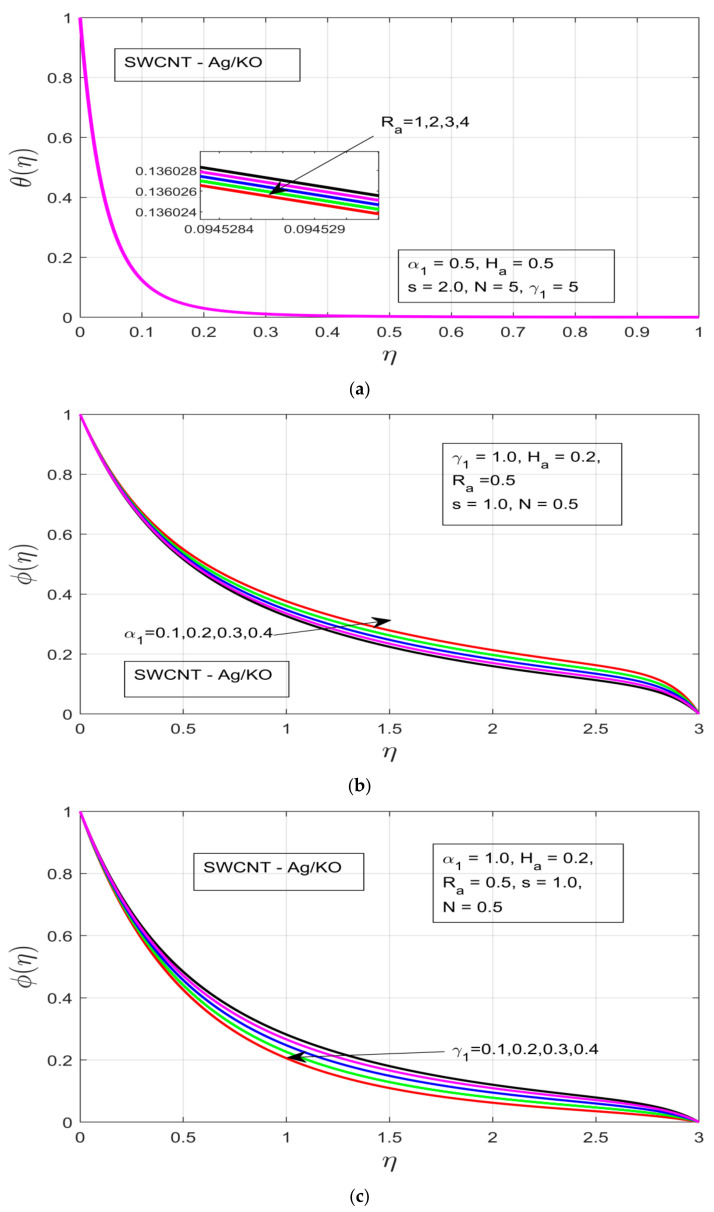

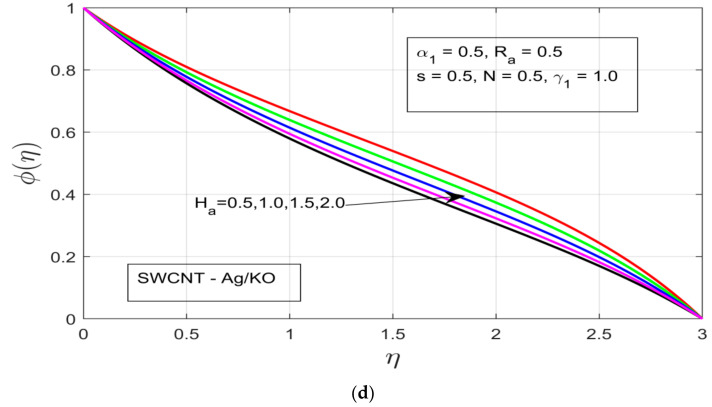
(**a**) Influence of the radiation *R_a_* on *θ(η)*. (**b**) Influence of the rotational ratio *α_1_* on *φ(η)*. (**c**) Effect of the buoyancy force *γ_1_* on *φ(η)*. (**d**) Influence of the Hartman number *H_a_* on *φ(η)*.

**Figure 6:**
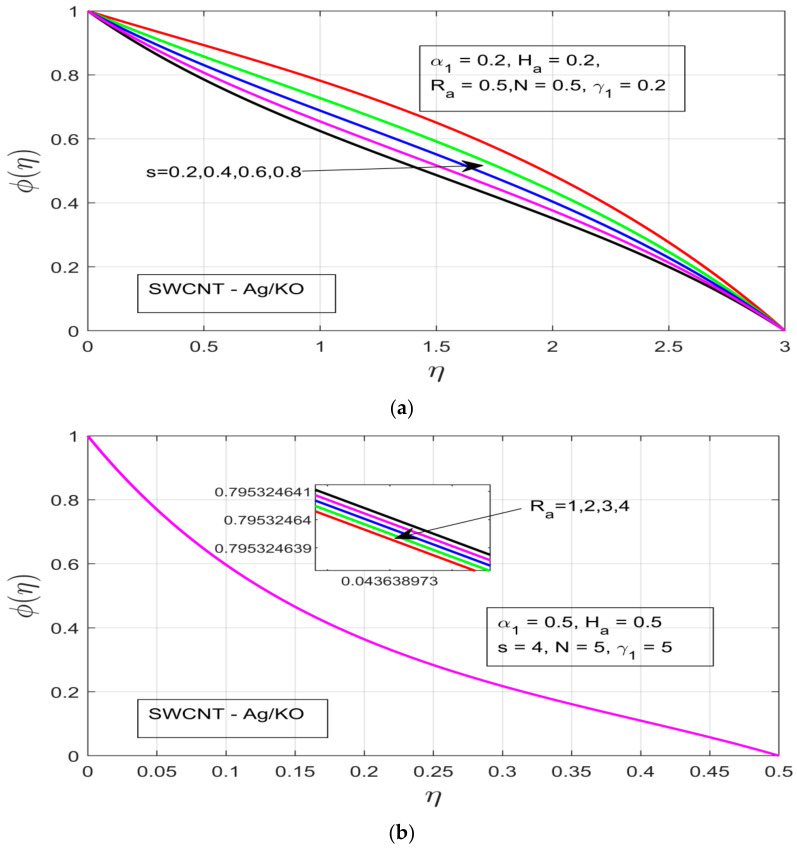

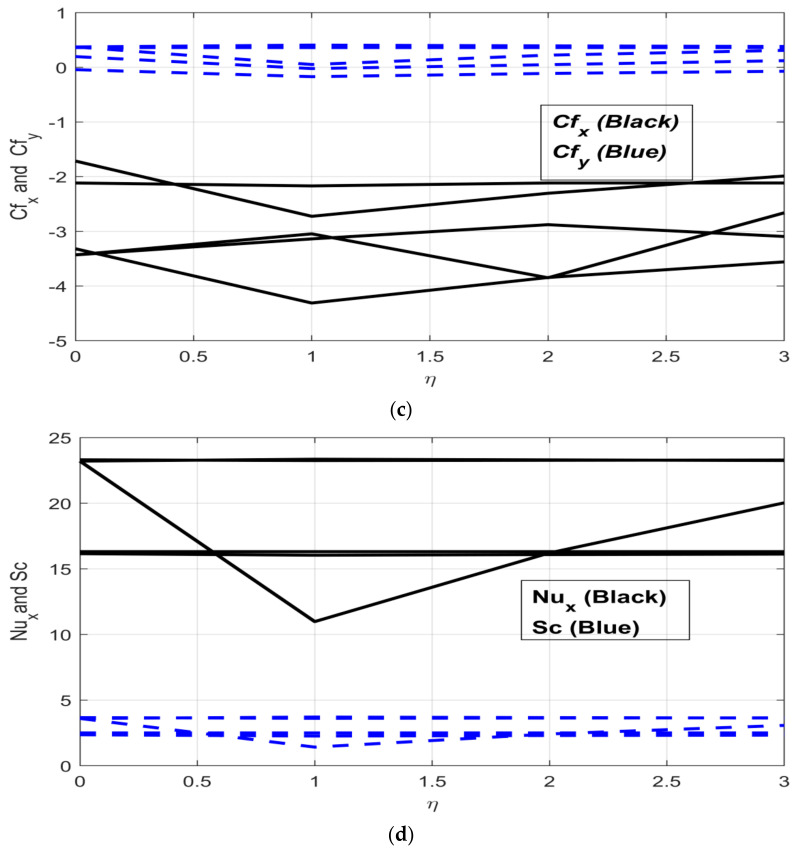
(**a**) Influence of the unsteadiness parameter *s* on the concentration profile *φ(η)*. (**b**) Influence of radiation *R_a_* on *φ(η)*. (**c**) Skin coefficients in x and y directions. (**d**) Heat and mass coefficients (*Nu_x_, Sc*).

Figure 5a–d demonstrates the influence of distinct study constraints on the behavior of the concentration profile. The radiative impression on the thermal boundary layer of the temperature profile can be observed in Figure 5a, where an applied magnetic field resistance force is created in the flow motion, which does not increase the thermal boundary—instead the thermal boundary layer contracts. Figure 5b illustrates the influence of the rotational ratio $\alpha_{1}$ on the concentration φ(η), whereby the concentration layer of the flow decreases as we increase the rotation $\alpha_{1}$ of the fluid. Figure 5c depicts the impact of the buoyancy force $\gamma_{1}$ on the concentration layer. The increasing pressure gradient decrease the concentration layer, which occurs due to the presence of a strong exerted pressure and high resistive force. Figure 5d and Figure 6a illustrate the behavior of $H_{a}$ and unsteadiness characterization parameters (s). Under both parameters the concentration profile φ(η) is decreased, which happens due to the presence of a strong resistive force, which ultimately results in the decline of the concentration layer of our hybrid nanofluid. Figure 6b demonstrates the concentration layer of the magnetized fluid. It can be observed that the concentration layer also contracts under the incremental influence of radiation parameters.

### 4.3. Skin Friction, Nusselt, and Sherwood Coefficients

The coefficients of drag force, termed “skin friction coefficients” $\left(Cf_{x},\;Cf_{y}\right)$, along with heat and mass coefficients $(Nu_{x},\;Sc;$ Nusselt and Sherwood numbers, respectively) are discussed in Figure 6c,d. The data for skin coefficients and heat and mass coefficients are provided in Table 1. Figure 6c shows the skin friction coefficients $\left(Cf_{x},\;Cf_{y}\right)$ under increasing study constraints. The skin coefficient $\left(Cf_{x}\right)\;$ decreases while the skin friction coefficient in the y-direction $\left(Cf_{y}\right)$ increases under the same increasing influence of study parameters $\left(\alpha_{1},\gamma_{1},\;H_{a},\;R_{a},\;\mathrm{and}\;s\right)$. Figure 6d depicts heat and mass transfer coefficients, where the increasing radiation $R_{a}$ has no impact on either the heat or mass transfer $\left(Nu_{x},\;Sc\right)$, as both stay consistent. Then, under increasing unsteadiness characterization of flow motion, both decrease to 0.5≤s<1, but both then increase again to 1.5≤s<2.

The increases in rotational flow ratio $\alpha_{1}$ and buoyancy parameter $\gamma_{1}$ (pressure gradient) have very minor impacts on the heat and mass transfer coefficients. Moreover, greater rates of heat and mass transfer were obtained under the increasing influence of rotational and buoyancy parameters. Ullah et al. [59] analyzed the generation of entropy for hybrid nanofluids (SWCNT-MWCNT–kerosene oil). The results they achieved were compared with those obtained for SWCNT–kerosene oil. The hybrid nanofluids showed great increases in heat transfer coefficient values. In the present study, instead of using multi-walled carbon nanotubes, we opted for hybrid nanofluids (SWCNT-Ag–kerosene Oil), and even better heat and mass transfer coefficients were obtained.

### 4.4. Nephograms of Velocity Profiles and Temperatures

**Figure 7:**
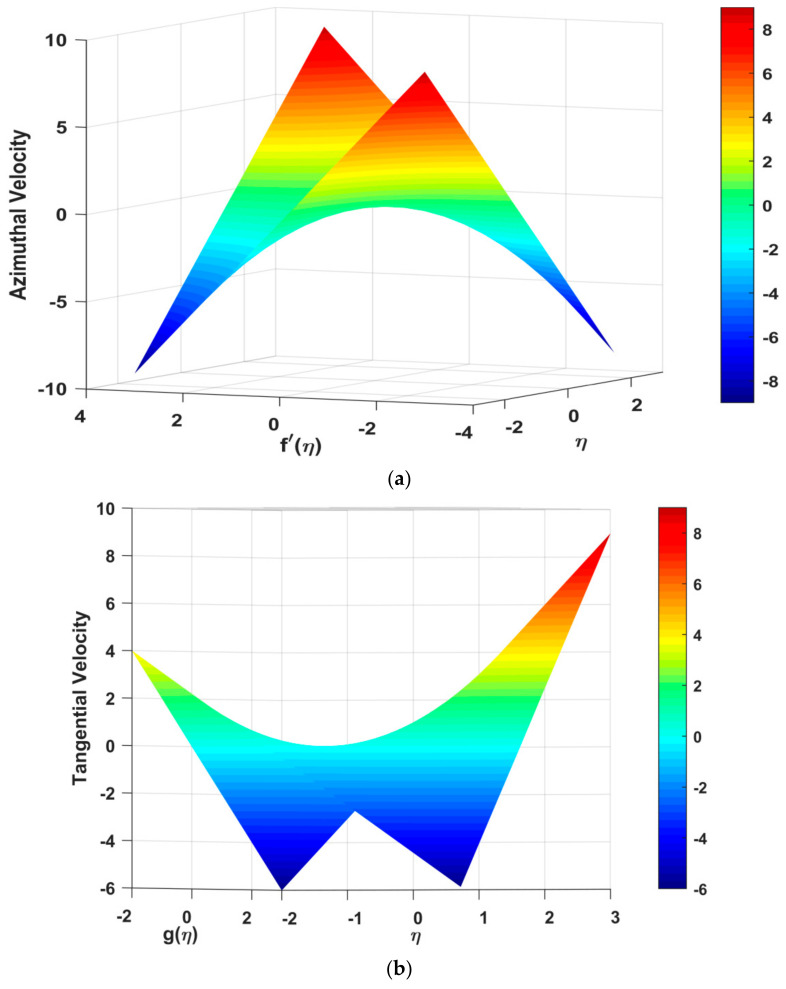

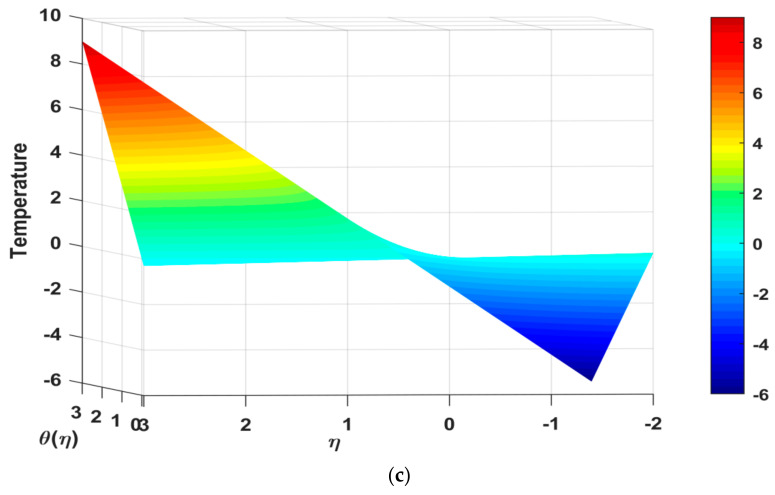
(**a**): Nephogram of *f′(η)* under the influence of the study parameters. (**b**) Nephogram of g(*η*) under the influence of the study parameters. (**c**) Nephogram of *θ(η)* under the influence of the study parameters.

The nephograms of velocity profiles and temperature profiles are depicted in Figure 7a–c, respectively. Figure 7a,b describes the azimuthal and tangential velocities. The tangential profile declines under increasing rotation influence, although it can also be observed when viewed in three dimensions that far at the boundary the fluid stream velocity slows down drastically (See Figure 7a). The tangential velocity can been seen to increase under the positively applied pressure gradient or buoyancy force. In Figure 7c, the temperature profile is illustrated, where the nephogram of the temperature shows that the temperature profile declines smoothly under the increasing impact of the study constraints (see Figure 7c).

## 5. Conclusions

In this article, the magneto-hydrodynamic hybrid nanofluid (SWCNT-Ag/KO) flow over an axially spinning cone was discussed. The prescribed wall temperature (PWT) was discussed for the heat transfer coefficient. The governing flow equations of the formulated problem were derived by utilizing the Rivlin–Ericksen tensor and boundary layer approximation. Numerical outcomes of the problem were drafted in MATLAB with the boundary value problem algorithm. The major outcomes of the study are as follows:

- The momentum boundary layer increased for the azimuthal velocity, accelerating the free stream fluid motion in the rotation parameter. The boundary layer declined under high-pressure force due to unsteadiness;
- The thickness of the flow boundary decreased under a positively applied pressure gradient (buoyancy force), expanded under rotation, and the magnetic field influenced the tangential velocity profile;
- Reduced skin friction coefficients for SWCNT-Ag/KO were observed under the increasing influence of different study constraints. These reduced skin rates for hybrid nanofluids were better than those for SWCNT/KO and Ag/KO;
- The mass conveyed with the SWCNT-Ag/KO hybrid nanofluid under the study constraints was high as compared to SWCNT-Ag/H_2_O. Due to unsteadiness in the flow movement, minimum rates of mass transfer were observed;
- The heat transfer coefficient (Nusselt number) for SWCNT-Ag/KO when compared with another hybrid nanofluid SWCNT-Ag/H_2_O was found to be considerably higher;
- The analyzed hybrid nanofluid (SWCNT-Ag–kerosene oil) produced minimum skin coefficient values and high thermal heat transfer rates when compared with a recent study for the SWCNT-MWCNT–kerosene oil hybrid nanofluid.

In general, the maximum Nusselt (Nu) and Sherwood (Sh) numbers were observed under a high rotational flow ratio and pressure gradient. The study suggests that more frequent use of the examined hybrid nanofluid would be beneficial. Moreover, the model can be useful in understanding characteristics of drug delivery, artery stenosis, and magnetized fluids in drug targeting during magneto-hyperthermia in the treatment of liver cancer.

## Figures and Tables

**Table 1:** The numerical values of skin friction coefficients *(Cf_x_*, *Cf_y_)* and Nusselt and Sherwood numbers *(Nu_x_*, *Sc).*

$\varphi_{1}$$=\varphi_{2}=0.005,$ *n* = 2, *Sc* = 2, *N* = 0.5, *pr* = 21								
					SWCNT-Ag/KO			
$\alpha_{1}$	$\gamma_{1}$	*R*	*H*	*s*	$Cf_{x}$	$Cf_{y}$	$Nu_{x}$	*Sc*
0.1	0.5	0.5	0.5	2	−1.71459	0.194793	23.3065	3.66768
0.2					−2.72506	−0.0238261	23.2472	3.6136
0.3					−2.30372	0.0470601	23.2694	3.63363
0.4					−1.98823	0.120427	23.2893	3.65172
0.5	0.5	0.5	1.0.	2	−3.43012	0.371631	23.2042	3.60143
	1				−1.04551	0.405135	23.3506	3.70912
	1.5				−1.84875	0.390947	23.3002	3.6694
	2				−2.66085	0.379969	23.2509	3.63317
0.5	2	1	2	1	−2.11565	0.359947	16.3115	2.5011
		2			−2.16934	0.359947	16.3115	2.5011
		3			−2.11565	0.359947	16.3115	2.5011
		4			−2.11565	0.359947	16.3115	2.5011
0.1	2	0.5	0.2	1	−3.31833	−0.0437809	16.1534	2.35923
			0.4		−4.31028	−0.172109	16.0379	2.26278
			0.6		−3.8436	−0.112958	16.085	2.30236
			0.8		−3.5578	−0.0728209	16.1223	2.33341
0.5	2	0.5	1.0.	0.5	−3.43012	0.371631	23.2042	3.60143
				1	−3.13779	0.0501056	10.9814	1.42014
				1.5	−2.87796	0.221208	16.2135	2.42122
				2	−3.09376	0.308887	20.0279	3.07409

**Table 2:** Different properties of the base fluid and nanoparticles utilized in the study [7,15,51,56,57].

Base Fluid	Nanoparticles	Thermophysical Properties		
		$\rho \;\left(Kgm^{-3}\right)$	$C_{p}\;\left(Kg^{-1}k^{-1}\right)$	$K\;\left(wm^{-1}k^{-1}\right)$
Kerosene Oil		783	2090	0.145
	Ag	10,490	235	429
	SWCNT	2600	425	600

**Table 3:** Thermophysical property relations for both kind of fluids. Reprinted from Ref. [7].

Properties	Nanofluid	Hybrid Nanofluid
Density (ρ)	$\rho_{nf}=\left(1-\varphi \right)*\rho_{f}+\varphi \rho_{s}$	$\rho_{hnf}=\rho_{f}\left(1-\varphi_{2}\right)\left[\left(1-\varphi_{1}\right)+\varphi_{1}\left(\frac{\rho_{s1}}{\rho_{f}}\right)\right]+\varphi_{2}\left(\frac{\rho_{s2}}{\rho_{f}}\right)$
Viscosity (μ)	$\mu_{nf}=\frac{\mu_{f}}{{\left(1-\varphi \right)}^{2.5}}$	$\mu_{hnf}=\frac{\mu_{f}}{{\left(1-\varphi_{1}\right)}^{2.5}{\left(1-\varphi_{2}\right)}^{2.5}}$
Heat capacity $\left(\rho c_{p}\right)$	${\left(\rho c_{p}\right)}_{nf}=\left(1-\varphi \right)*{\left(\rho c_{p}\right)}_{f}+\varphi *{\left(\rho c_{p}\right)}_{s}$	${\left(\rho C_{p}\right)}_{hnf}={\left(\rho C_{p}\right)}_{f}\left(1-\varphi_{2}\right)\left\{\left(1-\varphi_{1}\right)+\varphi_{1}\left[\frac{{\left(\rho C_{p}\right)}_{s1}}{{\left(\rho C_{p}\right)}_{f}}\right]\right\}\;\;\;\;\;\;\;+\varphi_{2}\left[\frac{{\left(\rho C_{p}\right)}_{s2}}{{\left(\rho C_{p}\right)}_{f}}\right]$
Thermal conductivity (k)	$\frac{k_{nf}}{k_{f}}=\frac{\left(k_{s}+2k_{f}\right)-2*\varphi *\left(k_{f}-k_{s}\right)}{\left(k_{s}+2k_{f}\right)+\varphi *\left(k_{f}-k_{s}\right)}$	$\frac{K_{hnf}}{k_{bf}}=\frac{K_{s2}+\left(n-1\right)K_{bf}-\left(n-1\right)\varphi_{2}\left(K_{bf}-K_{s2}\right)}{K_{s2}+\left(n-1\right)K_{bf}+\varphi_{2}\left(K_{bf}-K_{s2}\right)}$ $\frac{k_{nf}}{k_{f}}=\frac{K_{s1}+\left(n-1\right)K_{f}-\left(n-1\right)\varphi_{1}\left(K_{f}-K_{s1}\right)}{K_{s1}+\left(n-1\right)K_{f}+\varphi_{1}\left(K_{f}-K_{s1}\right)}$

**Table 4:** Comparison of the obtained outcomes with previously published work.

Comparison of Conventional Nanofluid (SWCNT–Kerosene Oil) and Hybrid Nanofluid (SWCNT-Ag–Kerosene Oil)				Comparison of Skin Coefficient		
		Shafiq et al. [56]	Present Work		Ameen et al. [57]	Present Work
R	$H_{a}$	$Nu_{x}$	$Nu_{x}$	$H_{a}$	$Cf_{x}$	$Cf_{x}$
0	0.3	6.25429	22.8364	0.5	−1.93809	−1.9244
0.2		7.3438	20.8304	0.6	−1.97461	−2.27983
0.4		7.62461	21.8364	0.7	−2.00896	−2.06492

**Table 5:** Comparison of obtained outcomes with previously published work.

$\mathrm{Comparison}\;\mathrm{of}\;\mathrm{Sherwood}\;\mathrm{Number}\;(Sh_{x})$			
*Sc* = 0.2	$H_{a}=0.2$	Joshi et al. [58]	Present Results
*n*	$\varphi_{1}=\varphi_{2}$	$Sh_{x}$	$Sh_{x}$
1	0.06	0.5006	0.538877
2		0.523504948	0.538566
3		0.545801582	0.538686
4		0.567581406	0.538788

## Data Availability

Data presented in this article are available on request from the corresponding author.

## Nomenclature

$B_{0}$	Magnetic field strength (NmA^−1^)	$Gr_{1},\;Gr_{2}$	Grashof Number for PWT case
$v_{e}$	Free stream velocity	$\gamma_{1},\;\gamma_{2}$	Buoyancy parameters for PWT
u, v, w	Components of velocity in x, y, and z(ms^−1^)	$\alpha^{*}$	Angle of rotation
T, C, D	Temperature, concentration, and mass diffusivity, respectively	N	The ratio of the Grashof number
$\omega_{1},\;\omega_{2}$	Cone and fluid’s angular velocities, respectively	$Re_{L}$	Reynolds number
ω	Sum of cone and fluid velocities	Pr	Prandtl number
$\zeta ,\;\zeta^{*}$	Volumetric coefficients of temperature and concentration expansion, respectively	Sc	Schmidt or Sherwood number
s	Unsteadiness in free stream velocity	$\rho_{hnf}$	Hybrid nanofluid density(Kgm^−3^)
PWT	Prescribed wall temperature	$K_{hnf}$	Hybrid nanofluid thermal conductivity(Wm^−1^K^−1^)
$\alpha_{1}$	Rotational ratio	${\left(\rho C_{p}\right)}_{hnf}$	Hybrid nanofluid heat capacity (Jkg^−1^ K^−1^)
*f,g*	Velocity profiles	θ, φ	Temperature and concentration profiles
$\sigma^{*}$	Stefan Boltzmann constant	$K^{*}$	Heat absorption coefficient
$\alpha_{1}$	Rotational ratio	$R_{a}$	Radiation parameter
*η*	Similarity variable	$\nu_{hnf}$	Hybrid nanofluid viscosity (mPa)
$k_{f}$	Base fluid’s thermal conductivity (Wm^−1^K^−1^)	$T_{\infty },\;C_{\infty }$	Temperature and concentration of free stream
$q_{r}$	Rosseland radiation	*PDE’s*	Partial differential equations
*SWCNT*	Single-walled carbon nanotube	*KO*	Kerosene oil
*L*	Characteristic length	*ODE’s*	Ordinary differential equations
*BLA*	Boundary layer approximation	*BVP*	Boundary value problem
*Ag*	Silver nanoparticle	ν	Dynamic viscosity
